# Dietary cinnamon promotes longevity and extends healthspan via mTORC1 and autophagy signaling

**DOI:** 10.1111/acel.14448

**Published:** 2025-01-06

**Authors:** Yuling Guo, Qing Zhang, Bi Zhang, Tong Pan, Elizabeth A. Ronan, Anthony Huffman, Yongqun He, Ken Inoki, Jianfeng Liu, X.Z. Shawn Xu

**Affiliations:** ^1^ College of Life Science and Technology, Key Laboratory of Molecular Biophysics of MOE Huazhong University of Science and Technology Wuhan Hubei China; ^2^ Life Sciences Institute, University of Michigan Ann Arbor Michigan USA; ^3^ Department of Molecular and Integrative Physiology University of Michigan Ann Arbor Michigan USA; ^4^ Department of Computational Medicine and Bioinformatics University of Michigan Ann Arbor Michigan USA; ^5^ Unit for Laboratory Animal Medicine University of Michigan Ann Arbor Michigan USA; ^6^ Bioland Laboratory Guangzhou Regenerative Medicine and Health Guangdong Laboratory Guangzhou China

**Keywords:** aging, *C. elegans*, lifespan, longevity

## Abstract

Cinnamon, renowned for its aromatic flavor, represents one of the most widely used spices worldwide. Cinnamon is also considered beneficial to human health with therapeutic potential for treating various diseases, ranging from diabetes and cancer to neurodegenerative diseases. However, the mechanisms underlying cinnamon's health benefits remain elusive. It is also unclear whether cinnamon has any role in aging. Using *C. elegans* as a model, here we show that feeding worms cinnamaldehyde (CA), the active ingredient in cinnamon oil, prolongs longevity. CA also promotes stress resistance and reduces β‐Amyloid toxicity in a *C. elegans* model of Alzheimer's disease. Mechanistically, CA exerts its beneficial effects through mTORC1 and autophagy signaling. Interestingly, CA promotes longevity by inducing a dietary restriction‐like state without affecting food intake, suggesting CA as a dietary restriction mimetic. In human cells, CA exerts a similar effect on mTORC1 and autophagy signaling, suggesting a conserved mechanism. Our results demonstrate that dietary cinnamon promotes both lifespan and healthspan and does so by regulating mTORC1 and autophagy signaling.

## Graphical abstract

Dietary cinnamon extends lifespan, enhances stress resistance, and ameliorates β‐Amyloid toxicity in *C. elegans* . Its effects are mediated by mTORC1 and autophagy signaling. Interestingly, cinnamon mimics the effect of dietary restriction without altering food intake. A similar effect of cinnamon on mTORC1 and autophagy was observed in human cells, suggesting a conserved mechanism.

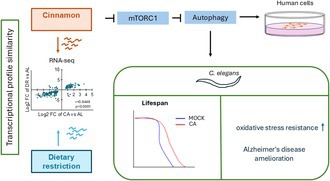

**Abbreviations**
AD: Alzheimer's disease
AITC: allyl isothiocyanate
AL: ad libitum
BECN1: beclin 1
CA: cinnamaldehyde
*C. elegans*
: Caenorhabditis elegans
DEG: differentially expressed gene
DESeq2: differential expression analysis for sequence count data 2
DR: dietary restriction
FDR: false discovery rate
FOXA: forkhead box A
FOXO: forkhead box O
GFP: green fluorescent protein
GO: gene ontology
HSF1: heat shock transcription factor 1
LC3: microtubule‐associated protein 1 light chain 3
mTOR: mammalian target of rapamycin
mTORC: mammalian target of rapamycin complex
NGM: nematode growth media
Nrf2: nuclear factor erythroid 2‐related factor 2
RAGA: ras related GTP binding A
Raptor: regulatory‐associated protein of mTOR
Rictor: raptor independent companion of mTOR
RNA‐seq: RNA sequencing
RSEM: RNA‐Seq by expectation maximization
SQSTM1: sequestosome 1
STAR: spliced transcripts alignment to a reference
TFEB: transcription factor EB
TRPA1: transient receptor potential A1
ULK1: autophagy‐related protein‐1 homolog unc‐51‐like kinase 1
WT: wild type

## 1 INTRODUCTION

Humans have an ancient history of using spices primarily for flavoring foods and beverages. Among commonly consumed spices, cinnamon is best known for its pleasant aroma and distinct taste, representing one of the most widely used spices worldwide, probably only second to black pepper (Report, 2018). Cinnamon is more than a flavorful spice. For example, cinnamon has also been recognized for its wide‐ranging health‐benefiting attributes, such as anti‐inflammatory, antibacterial, and antioxidant properties (Blaszczyk et al., 2021; Shu et al., 2024; Zhao et al., 2023). With such health‐benefiting features, cinnamon is considered possessing therapeutic potential for various human diseases (Banerjee & Banerjee, 2023; Momtaz et al., 2018; Shang et al., 2021; Silva et al., 2022). Indeed, it has been reported that cinnamaldehyde (CA), the active ingredient in cinnamon oil, can lower blood glucose and cholesterol levels, inhibit cancer cell proliferation, and is also neuroprotective (Medagama, 2015; Momtaz et al., 2018). As such, CA has been used to treat metabolic disorders (e.g., diabetes and obesity) and various cancers, as well as neurodegenerative diseases such as Alzheimer's disease (AD) (Banerjee & Banerjee, 2023; Momtaz et al., 2018; Shang et al., 2021; Silva et al., 2022). However, the mechanisms underlying cinnamon's health‐promoting effects are not well understood (Momtaz et al., 2018; Shang et al., 2021). In addition, the wide‐ranging health benefits of cinnamon bring the question of whether cinnamon has a role in aging; yet this has not been tested.

Here, we sought to address these questions in *C. elegans*, a well‐established genetic model organism for the biology of aging. We show that CA promotes longevity and stress resistance, as well as reduces β‐Amyloid toxicity in a *C. elegans* model of AD. CA exerts its beneficial effects on lifespan and healthspan via mTORC1 and autophagy signaling, a genetic pathway that is known to mediate dietary restriction (DR)‐induced longevity (Green et al., 2022; Kapahi et al., 2010; Kapahi et al., 2017). Strikingly, CA extends lifespan by inducing a DR‐like state in worms but without affecting their food intake, suggesting CA as a DR mimetic agent. Remarkably, CA also regulates mTORC1 and autophagy signaling in human cells, suggesting a common mode of action for CA in worms and mammals. Our findings reveal a previously unrecognized role of dietary cinnamon in aging and identify a mechanistic basis underlying cinnamon's health‐benefiting properties.

## 2 RESULTS

### 2.1 Dietary CA extends lifespan in a dose‐dependent manner

**FIGURE 1:**
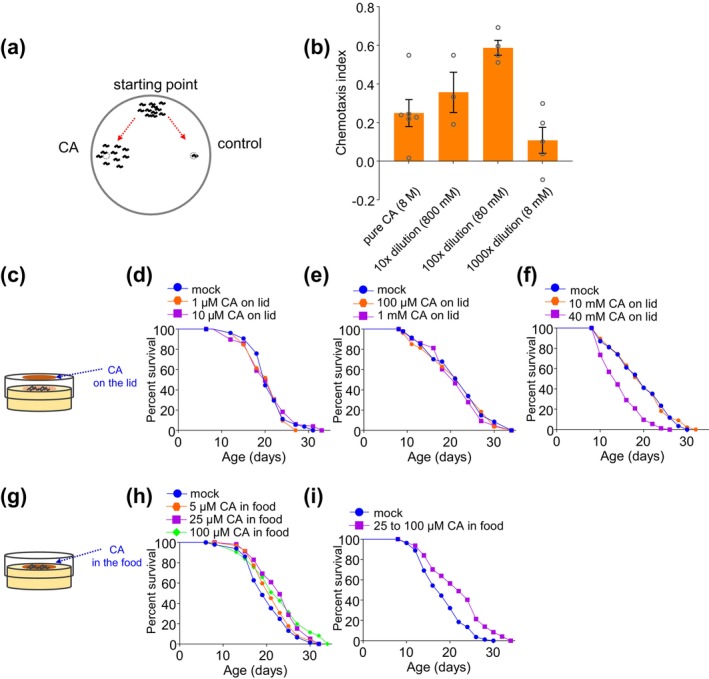
Dietary CA extends lifespan in *C. elegans*. (a, b) Worms are attracted to CA odor. (a) A schematic diagram describing the chemotaxis assay. Briefly, 50–100 day 1 adult worms were collected, washed to remove bacteria, and then placed on the starting point to test chemoattraction to the odorant in the dark for one hour. The chemotaxis index was calculated as [N_(attractant)_‐N_(counterattractant)_]/ [N_(attractant)_ + N_(counterattractant)_]. (b) Bar graph showing the chemotaxis index of CA of various concentrations. Four concentrations were tested, including 8, 80, 800 mM and pure CA. Data is presented as mean ± SEM. Experiments were repeated at least three times. (c–f) CA odor does not extend lifespan. (c) A schematic diagram depicting the lifespan assay. A chunk of agar absorbed with CA (50 μL) of varying concentrations was placed on the lid. Worms were treated with CA odor from the L4 stage. (d‐f) Different concentrations of CA odor do not extend lifespan (*p* = 0.565 for 1 μM, 0.739 for 10 μM, 0.717 for 100 μM, 0.282 for 1 mM, 0.74 for 10 mM, respectively, all against mock control), and the odor of 40 mM CA even shortened lifespan, *p* < 0.0001. Worms were treated with odor from the L4 stage and transferred every other day until day 12, at which point no more progeny was generated (f). (g–i) Dietary CA extends lifespan. (g) A schematic diagram depicting the lifespan assay. CA was included in bacteria food on the plate as a food supplement. Worms were treated with CA from the L4 stage and transferred every other day until day 12, at which point no more progeny was generated. (h) Diverse concentrations of dietary CA extend lifespan to different levels (*p* = 0.178 for 5 μM, 0.002 for 25 μM, 0.003 for 100 μM, respectively, all against mock control). The mean lifespan of worms fed 25 μM CA was slightly longer than that of 100 μM (23.19 ± 0.64 vs. 22.77 ± 0.80), while the latter achieved a greater maximum lifespan (32‐days vs. 34‐days). (i) The lifespan assay was optimized by first feeding worms 25 μM of CA (till day 12) followed by 100 μM of CA, *p* < 0.001. Kaplan–Meier survival analysis with log‐rank test was used for statistical analysis. See Table S1 for lifespan statistics.

Cinnamon features a distinct aromatic smell. As odors from food have a profound effect on lifespan (Park et al., 2021; Zhang et al., 2021), we wondered if cinnamon odor regulates longevity. The distinct aroma of cinnamon is primarily derived from CA, the active ingredient in cinnamon oil (Blaszczyk et al., 2021). We thus focused on testing the odor from CA. Indeed, we found that worms were attracted to diverse concentrations of CA odor, shown by a two‐choice chemotaxis assay (Figure 1a,b). This prompted us to question if the odor itself regulates lifespan. To test this possibility, we adopted a lifespan assay in which we included CA on the lid of NGM plates such that worms were exposed to CA odor but not in direct contact with CA (Figure 1c) (Park et al., 2021; Zhang et al., 2021). Unexpectedly, CA odor did not extend lifespan (Figure 1d–f). In fact, CA odor even shortened lifespan at high concentrations (e.g., 40 mM) (Figure 1f). Apparently, CA odor does not appear to have a beneficial impact on longevity.

We thus tested the effect of CA itself on lifespan by feeding CA directly to worms. To do so, we included CA on NGM plates as a food supplement and found that dietary CA extended lifespan (Figure 1g–i). Notably, lower concentrations of CA (e.g., 5 μM and 25 μM) were more efficient in extending lifespan in early‐middle life, while higher concentrations of CA (e.g., 100 μM) achieved a better effect in late life with a longer maximal lifespan but a slightly shorter mean lifespan compared to that under the former condition (Figure 1h). No lifespan extension was observed when we further increased the CA concentration (e.g., 300 μM) (Figure S1a,b), indicating a dose‐dependent effect of CA. We then optimized the lifespan assay by feeding worms 25 μM of CA in early‐middle life followed by 100 μM of CA in late life (Figure 1i). As a control, CA treatment did not have a notable effect on worm brood size, body size, or locomotion, though it increased lipid storage (Figure S1g–j). Taken together, dietary CA appears to extend lifespan in a dose‐dependent manner.

High concentrations of CA have been reported to inhibit bacteria growth (Mohamed et al., 2020; Shan et al., 2007). For example, CA at the mM range can inhibit the growth of *E. coli* bacteria (Shan et al., 2007). As we fed worms *E. coli* bacteria (OP50), it might be argued that inhibition of bacteria growth by CA would mimic a dietary restriction (DR) effect, thereby extending lifespan. However, CA did not affect the growth rate of OP50 bacteria at concentrations (i.e., 25–100 μM) used in our lifespan assay (Figure S1c), arguing against this possibility. Similar to the case with live bacteria, worms fed carbenicillin‐killed bacteria containing CA also lived a longer lifespan (Figure S1d). These control experiments suggest that CA extends lifespan as a food additive rather than by affecting bacteria growth.

### 2.2 CA promotes longevity in a TRPA‐1‐independent manner

How does cinnamon extend lifespan? Previous studies have shown that TRPA1 channel is a target of CA (Bandell et al., 2004; Jordt et al., 2004). The *C. elegans* TRPA1 homolog TRPA‐1 extends lifespan at low temperatures (Xiao et al., 2013; Zhang et al., 2015; Zhang et al., 2018), raising the possibility that CA might promote longevity by activating TRPA‐1. However, CA can promote longevity in *trpa‐1* mutant worms (Figure S1e), indicating that CA acted independently of TRPA‐1. To provide additional evidence, we tested the effect of the TRPA1 agonist AITC on lifespan (Jordt et al., 2004), and found that unlike CA, feeding worms AITC did not extend lifespan (Figure S1f). Taken together, these results demonstrate that CA promotes longevity in a TRPA1‐independent manner in worms, suggesting that CA acts through a previously unknown mechanism in lifespan regulation.

### 2.3 CA‐induced lifespan extension requires PHA‐4/FOXA

**FIGURE 2:**
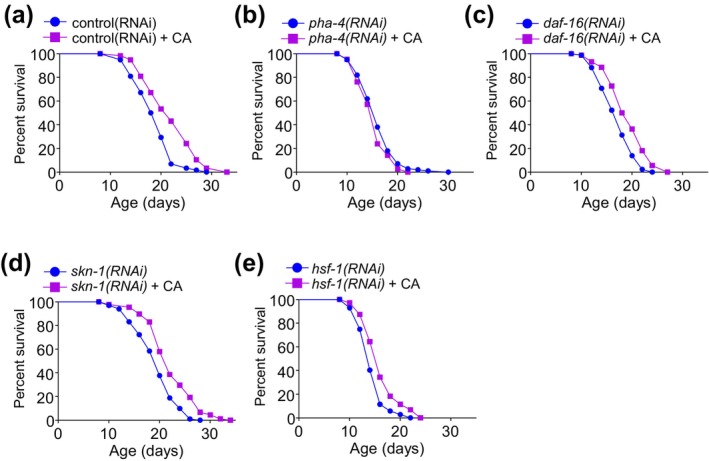
CA‐induced lifespan extension depends on PHA‐4/FOXA. (a, b) CA‐induced lifespan extension requires *pha‐4/FOXA*. (a) Control (empty vector) RNAi, *p* < 0.001. (b) RNAi knockdown of *pha‐4* abolishes CA‐induced longevity, *p* = 0.079. (c–e) CA still extends lifespan in worms treated with RNAi against *daf‐16/FOXO, p* < 0.001 (c), *skn‐1/Nrf2, p* < 0.001 (d), or *hsf‐1/HSF1, p* = 0.044 (e). Worms were treated with RNAi and 25 μM CA from the L4 stage and transferred every other day until day 12, at which point CA concentration was increased to 100 μM and no more progeny was generated. See Methods for details. Kaplan–Meier survival analysis with log‐rank test was used for statistical analysis. See Table S1 for lifespan statistics.

Longevity pathways tend to converge on a subset of transcription factors (Kenyon, 2010). Thus, as a first step toward the elucidation of the mechanisms underlying CA longevity, we asked which transcription factor(s) is necessary for CA to extend lifespan. We found that the FOXA transcription factor PHA‐4 was required for CA longevity, as CA can no longer extend lifespan in worms deficient in *pha‐4* (Figure 2a,b; Figure S2a,b). By contrast, CA can still extend lifespan in worms deficient in several other transcription factors such as *daf‐16/FOXO, skn‐1/Nrf2*, and *hsf‐1/HSF1* (Figure 2c–e and Figure S2c–e). This indicates that though other transcription factors might also be involved, the FOXA transcription factor PHA‐4 plays an essential role in mediating CA longevity.

### 2.4 CA‐induced lifespan extension depends on mTORC1 and autophagy signaling

**FIGURE 3:**
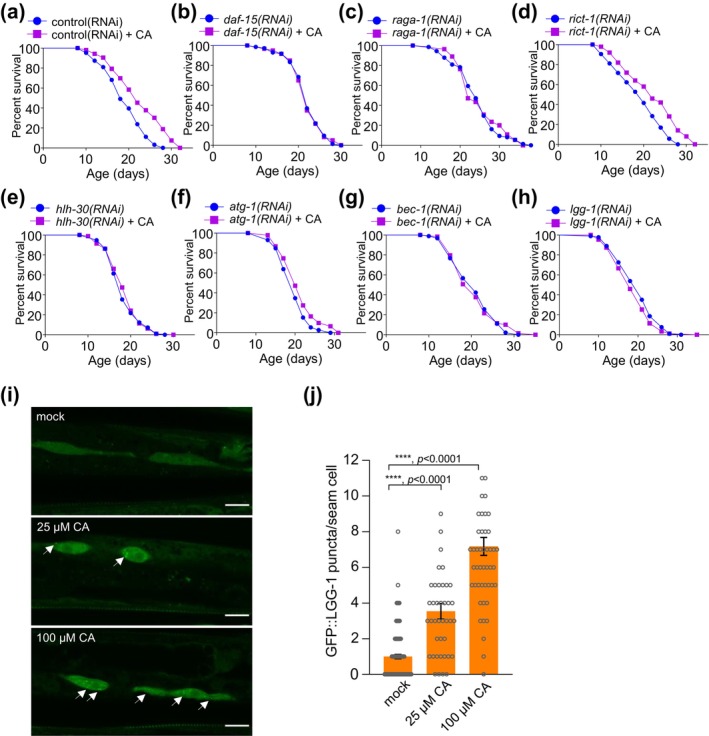
Dietary CA extends lifespan via mTORC1 and autophagy signaling. (a–d) Inhibition of mTORC1 signaling abolishes CA‐induced longevity. RNAi knockdown of *daf‐15/Raptor, p =* 0.966 (b) and *raga‐1/RAGA*, *p* = 0.805 (c), but not *rict‐1/RICTOR, p* = 0.002 (d), eliminates the lifespan extension effect of CA, *p* < 0.001 (a). (e‐h) Inactivation of autophagy signaling inhibits CA‐induced longevity. RNAi knockdown of *hlh‐30/TFEB, p* = 0.513 (e), *atg‐1/ULK1, p* = 0.011 (f), *bec‐1/BECN1, p* = 0.715 (g), and *lgg‐1/LC3, p* = 0.159 (h) inhibits the lifespan extension effect of CA. Worms were treated with RNAi and 25 μM CA from the L4 stage and transferred every other day until day 12, at which point CA concentration was increased to 100 μM and no more progeny was generated. See Methods for details. Kaplan–Meier survival analysis with log‐rank test was used for statistical analysis. (i‐j) CA promotes autophagy. (i) Sample images showing that dietary CA increases the number of LGG‐1::GFP puncta in seam cells of L3/4 worms. 10–15 worms were assayed for each experiment, and the figure included data points for all three biological independent experiments. Fluorescence was visualized under an Olympus confocal microscope with a 40X objective lens. Z stack images of hypodermal seam cells were captured, and maximum intensity Z‐projection images were used for quantification. GFP::LGG‐1 puncta were quantified by manual counting. Arrows point to LGG‐1::GFP puncta. Scale bar: 10 μm. (j) Bar graph quantifying the data in (I). Data is presented as mean ± SEM. Experiments were repeated at least three times. *P* values were calculated using one‐way ANOVA with Dunnett's test. The statistical analysis of HT115 control was shown in Table S1, *p* < 0.001. See Table S1 for lifespan statistics.

PHA‐4 is known to act downstream of mTOR to regulate lifespan (Sheaffer et al., 2008). Hence, we examined if mTOR contributes to CA longevity. mTOR forms two evolutionarily conserved protein complexes, mTORC1 and mTORC2, which are distinguished from each other by their specific subunits (e.g., Raptor for mTORC1 and Rictor for mTORC2), and their regulatory proteins and substrates (e.g., RAGA for mTORC1) (Kim & Guan, 2019; Saxton & Sabatini, 2017; Wullschleger et al., 2006). Inactivation of the mTORC1‐specific components DAF‐15/Raptor and RAGA‐1/RAGA abolished the ability of CA to extend lifespan (Figure 3a–c; Figure S2f), while worms deficient in RICT‐1/Rictor, a specific component of mTORC2, remained sensitive to CA (Figure 3d; Figure S2g). Thus, mTORC1 rather than mTORC2 appears to be required for CA longevity.

PHA‐4/FOXA modulates aging primarily by regulating autophagy and does so by acting downstream of mTORC1 (Deleyto‐Seldas & Efeyan, 2021; Hansen et al., 2008; Panowski et al., 2007). In addition, autophagy is known to reside downstream of most, if not all, longevity pathways (Hansen et al., 2018; Kenyon, 2010; Nakamura & Yoshimori, 2018). This suggests that CA may promote longevity through the mTORC1‐PHA‐4/FOXA‐autophagy signaling pathway, prompting us to hypothesize that autophagy may be involved in CA longevity. To test this, we examined HLH‐30/TFEB, another autophagy‐regulating transcription factor (Hansen et al., 2018; Nakamura & Yoshimori, 2018; Settembre et al., 2011). PHA‐4/FOXA and HLH‐30/TFEB act cooperatively to regulate autophagy by controlling the expression of autophagy‐ and lysosome‐related genes (Klionsky et al., 2021; Lim et al., 2023). Inactivation of *hlh‐30/TFEB* by RNAi abolished CA longevity (Figure 3e; Figure S2h). Similarly, RNAi inactivation of *atg‐1*/*ULK1*, another autophagy‐regulating gene (Klionsky et al., 2021; Mizushima, 2010), also severely compromised CA longevity (Figure 3f). Notably, PHA‐4/FOXA, HLH‐30/TFEB and ATG‐1/ULK1 are all known to act downstream of mTORC1 to regulate autophagy (Kenyon, 2010; Kim & Guan, 2019; Saxton & Sabatini, 2017; Wullschleger et al., 2006). These results all point to a key role of autophagy in CA longevity. Encouraged by these observations, we directly tested a possible involvement of autophagy in CA longevity by examining BEC‐1/BECN1 and LGG‐1/LC3, two essential components of the autophagy machinery (Hansen et al., 2018; Klionsky et al., 2021). Indeed, inactivation of *bec‐1* and *lgg‐1* by RNAi abolished the ability of CA to extend lifespan (Figure 3g,h), revealing an essential role of autophagy in CA longevity. We thus conclude that autophagy is required for CA to extend lifespan.

To investigate how CA promotes longevity through autophagy signaling, we quantified autophagic flux by imaging LGG‐1::GFP puncta, a commonly used assay to measure autophagy signaling in *C. elegans* (Kang et al., 2007; Klionsky et al., 2021). The number of GFP::LGG‐1 puncta was significantly increased in worms fed CA (Figure 3i,j), indicating that CA can stimulate autophagy. These data together suggest that CA extends lifespan by promoting autophagy. Our results also suggest a model in which CA longevity is mediated by mTORC1‐autophagy signaling.

### 2.5 Dietary CA promotes stress resistance in an mTORC1 and autophagy‐dependent manner

**FIGURE 4:**
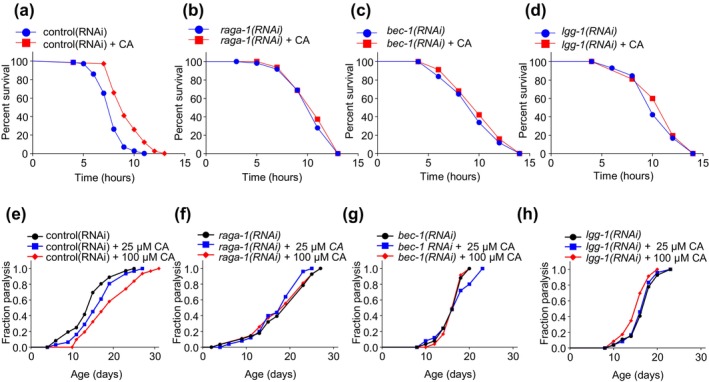
Dietary CA promotes stress resistance and ameliorates β‐Amyloid toxicity in an mTORC1‐ and autophagy‐dependent manner. (a–d) Time course survival curve of worms that were fed 100 μM CA for 2 days (L4 to day 2) and then exposed to 9.125 mM TBHP. Thirty worms were assayed for each experiment. Survival was scored every 2 h. (a) Dietary CA enhances oxidative stress resistance, *p* < 0.0001. (b–d) The enhanced stress resistance induced by CA is lost after RNAi knockdown of *raga‐1/RRAGA* (b), *bec‐1/BECN1* (c) and *lgg‐1/LC3* (*p* = 0.38, 0.306, 0.276, respectively) (d). (e–h) Time course of β‐Amyloid induced paralysis in worms fed various concentrations of CA (25 μM, 100 μM). Animals were treated with RNAi and CA from L4, and about 30 worms were assayed for each experiment. Paralysis was scored every day or every other day until all animals were paralyzed. Dietary CA slows down the age‐dependent paralysis phenotype, *p* < 0.0001 for both 25 μM and 100 μM (e). RNAi knockdown of *raga‐1/RRAGA, p* = 0.057, 0.503 for 25 μM and 100 μM respectively (f), *bec‐1/BECN1, p* = 0.565, 0.59 for 25 μM and 100 μM respectively (g) and *lgg‐1/LC3, p* = 0.588, 0.027 for 25 μM and 100 μM respectively (h) abolish the beneficial effect of CA. Kaplan–Meier survival analysis with log‐rank test was used for statistical analysis. See Table S1 for healthspan statistics.

Lifespan extension is usually, though not always, accompanied by an extension of healthspan (Fontana et al., 2010). As animals age, they become more vulnerable to stresses (Lopez‐Otin et al., 2023). Stress resistance has thus been commonly utilized as a measure for healthspan (Kenyon, 2010). To test if CA can also extend healthspan, we examined CA‐treated worms for their resistance to oxidative and heat stresses. Though worms fed CA exhibited similar resistance to heat (Figure S2j), CA treatment enhanced their resistance to oxidative stress (Figure 4a; Figure S2j), indicating that dietary CA promotes oxidative stress resistance. Importantly, such enhanced stress resistance was lost in worms deficient in *raga‐1, bec‐1*, and *lgg‐1* (Figure 4b–d), indicating that CA promotes oxidative stress resistance through mTORC1 and autophagy signaling, which is similar to the case with CA longevity. It thus appears that dietary CA promotes both lifespan and healthspan via mTORC1 and autophagy signaling.

### 2.6 Dietary CA ameliorates β‐amyloid toxicity

Aging is recognized as a primary risk factor for the development of neurodegenerative diseases, particularly Alzheimer's disease (AD) (Hou et al., 2019). As dietary CA delays aging, we asked if CA can protect against β‐Amyloid toxicity. To test this, we took advantage of a commonly used worm model of AD (Link, 1995), in which transgenic expression of human β‐amyloid peptide leads to an age‐dependent progressive paralysis phenotype caused by the toxicity of β‐Amyloid aggregates. Strikingly, dietary CA slowed down such age‐dependent paralysis in these transgenic worms (Figure 4e), suggesting that CA can ameliorate β‐Amyloid toxicity. Importantly, CA‐induced protection against β‐Amyloid toxicity was abolished in *raga‐1* RNAi worms (Figure 4f), indicative of a requirement for mTORC1 in this process. A similar phenomenon was observed in *bec‐1* and *lgg‐1* RNAi worms (Figure 4g,h), pointing to an essential role of autophagy in CA‐induced protection against β‐Amyloid toxicity. These results demonstrate that dietary CA ameliorates β‐Amyloid toxicity via mTORC1 and autophagy signaling.

### 2.7 Dietary CA induces a DR‐like state in worms without affecting their food intake

**FIGURE 5:**
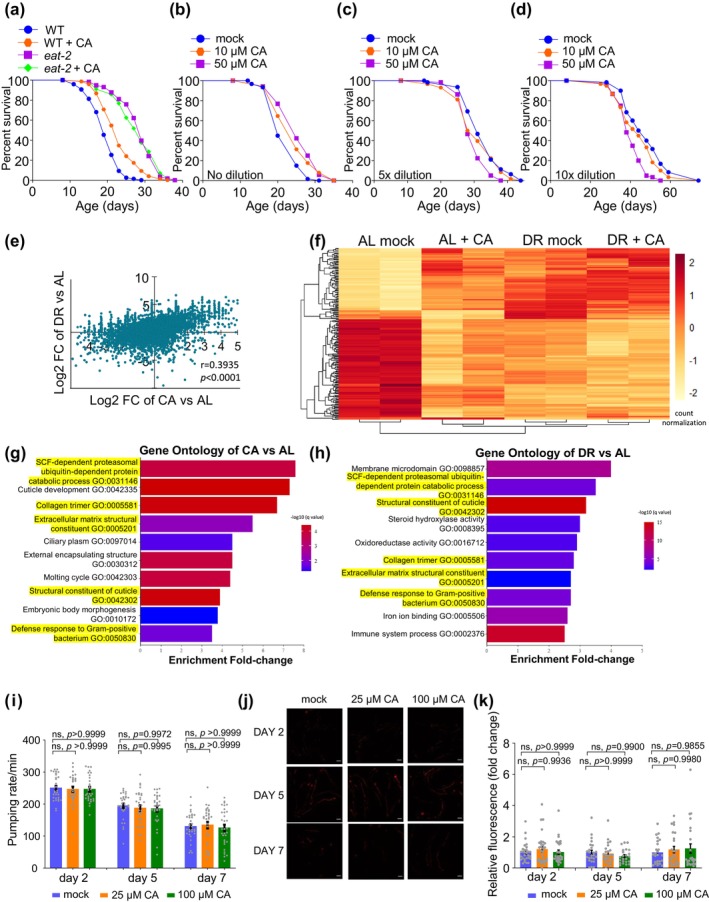
CA induces a DR‐like state in worms without affecting food intake. (a) CA can no longer extend lifespan in *eat‐2(ad465)* mutant worms, *p* = 0.938. Worms were treated with 25 μM CA from the L4 stage and transferred every other day until day 12, at which point CA concentration was increased to 100 μM and no more progeny was produced. Kaplan–Meier survival analysis with log‐rank test was used for statistical analysis. (b–d) Lifespan survival curves of worms under bDR. The concentration of CA was optimized again because the assay was switched from solid plates to liquid culture. See Methods for details. (b) CA extends the lifespan of worms fed AL (10^10^ cfu/mL) at both concentrations (*p* = 0.003 for 10 μM and *p* < 0.001 for 50 μM of CA). (c, d) CA cannot extend the lifespan of worms under bDR: 5‐fold dilution of food (*p* = 0.188 for 10 μM and *p* < 0.001 for 50 μM) in (c), and 10‐fold dilution of food (*p* = 0.091 for 10 μM and *p* < 0.001 for 50 μM) in (d). (e) Two‐axis scatter plot of log2‐fold changes showing the expression fold change of genes across the whole genome. Each dot represents the expression log2‐fold change of CA vs. AL (X‐axis value) and the expression log2‐fold change of DR vs. AL (y‐axis value). Pearson correlation coefficient (*r*) and *p* value were shown. (f) Hierarchical clustering of DEGs. The analysis was performed based on the 205 DEGs genes that were shared between CA and DR treatments. (g, h) The top 10 most enriched gene ontology (GO) terms of genes differentially expressed in CA vs. AL (g) and DR vs. AL (h). FDR < 0.1. Half of them overlap with each other (highlighted in yellow). (i) Dietary CA does not alter the pumping rate of worms. Data is presented as mean ± SEM. Experiments were repeated at least three times. *P* values were calculated using one‐way ANOVA with Dunnett's test. (j, k) Dietary CA does not alter the amount of bacterial food ingested by worms. (j) Sample images. Worms were fed bacterial food mixed with fluorescence beads for one hour before imaging. Z stack images of the intestine were captured, and maximum‐intensity Z‐projection images were used for quantification. Scale bar: 100 μm. (k) Bar graph showing normalized fluorescence fold change. Data is presented as mean ± SEM. Experiments were repeated at least three times. *P* values were calculated using one‐way ANOVA with Dunnett's test. See Table S1 for lifespan statistics. See Table S2 for gene expression data from RNA‐seq and gene ontology analysis.

mTORC1‐autophagy signaling is well known to mediate dietary restriction (DR)‐induced longevity (Kapahi et al., 2010; Kapahi et al., 2017; Panowski et al., 2007). This, together with our observation that mTORC1‐autophagy signaling also mediates CA longevity, led us to postulate that DR and dietary CA might share some common features. As a first step to test this hypothesis, we examined *eat‐2* mutant worms, a genetic model of DR (Lakowski & Hekimi, 1998; Raizen et al., 1995). While dietary CA extended lifespan in wild‐type worms, it failed to do so in *eat‐2* mutant worms (Figure 5a), suggesting that CA and DR may act in the same or overlapping pathway. To garner additional data, we tested another DR model (bDR) by directly diluting bacterial food fed to worms (Greer & Brunet, 2009; Kapahi et al., 2017; Mair et al., 2009). Similarly, dietary CA failed to promote the lifespan of worms under DR, though it extended the lifespan of those fed ad libitum (AL) (Figure 5b–d). Interestingly, higher concentrations of CA not only did not further extend the lifespan of worms under DR, but even slightly shortened it (Figure 5d). Together, these results support the model that dietary CA and DR may act in the same or overlapping pathway, suggesting that these two regimens may share some common features.

To further characterize the effect of dietary CA and its relationship with DR, we conducted transcriptomic analyses of worms fed CA or DR by RNA‐seq. The data uncovered a conspicuous correlation between the differentially expressed genes (DEGs) induced by CA (CA vs. AL) and by DR (DR vs. AL) (Figure 5e,f; Figure S3). There is also a substantial overlap between DEGs regulated by CA and DR (Figure 5f; Figure S3; Table S2). Furthermore, Gene Ontology (GO) analysis showed that among the top 10 GO terms, half of them (5/10) were shared by the worms treated with CA and DR (Figure 5g,h), revealing a substantial overlap. The similarities between the transcriptome of worms fed CA and under DR are consistent with the model that dietary CA and DR may impinge on overlapping or similar genes/pathways, suggesting that dietary CA induces a DR‐like state.

One main characteristic of animals under DR is the decrease in food intake (Kapahi et al., 2017). We thus questioned if CA extends lifespan by reducing food intake as does DR. To test this possibility, we examined the rate of pharyngeal pumping as well as the amount of food ingested into the worm gut. No notable difference was detected between the worms fed CA and the control group (Figure 5i–k), indicating that CA did not affect food intake. Taken together, our data show that dietary CA induces a DR‐like state in worms without altering food intake, suggesting that CA may act as a DR mimetic.

### 2.8 CA inhibits mTORC1 and promotes autophagy in human cells

**FIGURE 6:**
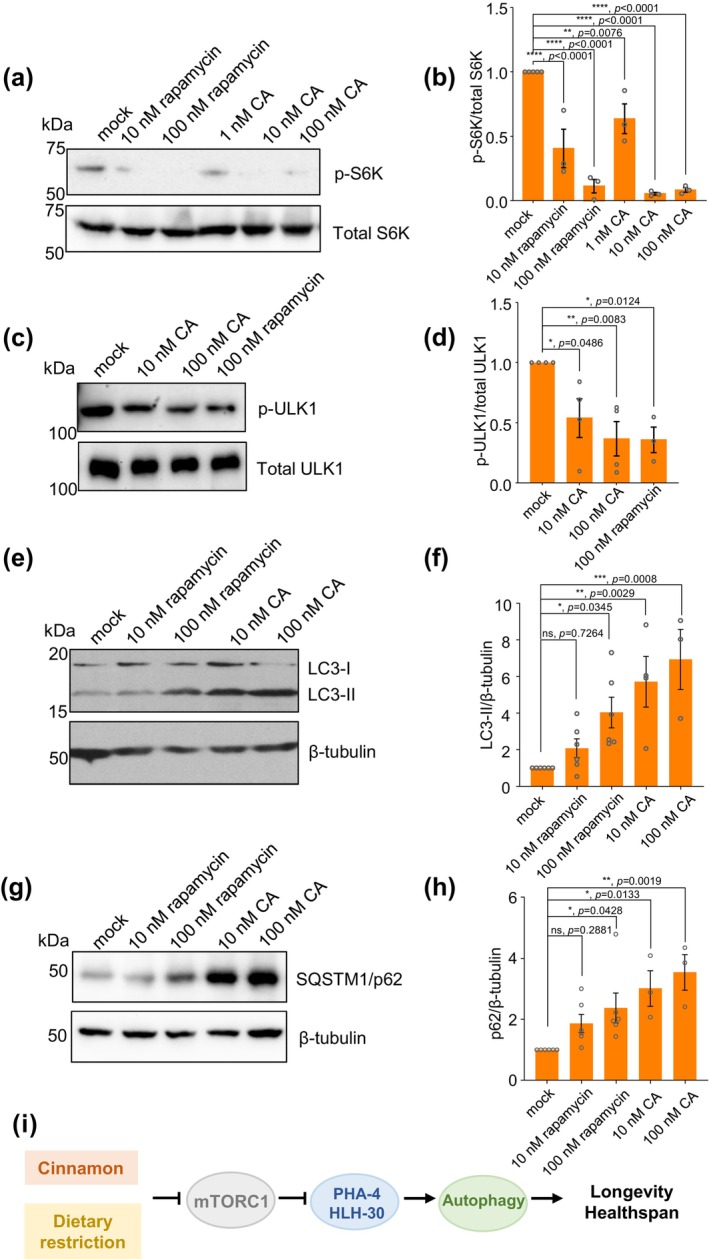
CA inhibits mTORC1 and promotes autophagy in HEK293T cells. (a, b) CA inhibits the phosphorylation level of S6K (ribosomal S6 kinase) in HEK293T cells. Rapamycin, an mTORC1 inhibitor, was used as a positive control. (a) Western blotting showing that CA inhibits the phosphorylation of Thr389 site in S6K. (b) Bar graph. Data is presented as mean ± SEM. Experiments were repeated at least 3 times. *P* values were calculated using one‐way ANOVA with Dunnett's test. (c, d) CA inhibits the phosphorylation level of ULK1 in HEK293T cells. Rapamycin, an mTORC1 inhibitor, was used as a positive control. (c) Western blotting showing CA inhibits the phosphorylation of Ser757 site in ULK1. (d) Bar graph. Data is presented as mean ± SEM. Experiments were repeated at least 3 times. *P* values were calculated using one‐way ANOVA with Dunnett's test. (e, f) CA enhances the LC3‐II level. Rapamycin was used as a positive control. (e) Western blotting showing that CA increases the amount of LC3‐II in the presence of E‐64d (10 nM) and leupeptin (100 nM). (f) Bar graph. Data is presented as mean ± SEM. Experiments were repeated at least 3 times. *P* values were calculated using one‐way ANOVA with Dunnett's test. (g, h) CA increases the SQSTM1/p62 level. Rapamycin was used as a positive control. (g) Western blotting showing that CA increases the amount of SQSTM1/p62 in the presence of E‐64d (10 nM) and leupeptin (100 nM). (h) Bar graph. Data is presented as mean ± SEM. Experiments were repeated at least 3 times. *P* values were calculated using one‐way ANOVA with Dunnett's test. (i) Schematic model.

As mTORC1 inhibits autophagy, inhibition of mTORC1 then activates autophagy (Deleyto‐Seldas & Efeyan, 2021; Kim & Guan, 2019; Saxton & Sabatini, 2017; Wullschleger et al., 2006). This mTORC1‐autophagy signaling axis is highly conserved in eukaryotes (Kim & Guan, 2019; Saxton & Sabatini, 2017; Wullschleger et al., 2006). Our observation that dietary CA acts through mTORC1‐autophagy signaling in *C. elegans* raises the possibility that a similar phenomenon might occur in mammals. We thus tested if CA can inhibit mTORC1 activity and stimulate autophagy in mammalian cells. We first assessed the effect of CA on mTORC1 activity by monitoring the level of Thr389 phosphorylation in S6K, a direct substrate of mTORC1 (Saxton & Sabatini, 2017). Indeed, CA treatment inhibited S6K phosphorylation in HEK293T cells (Figure 6a,b), indicating that CA can inhibit mTORC1 activity in these cells. We tested rapamycin as a control and found that as expected, it inhibited mTORC1 (Figure 6a,b). CA inhibition of mTORC1 was also observed in HeLa cells (Figure S4). As CA inhibited mTORC1 at a higher efficacy in HEK293T cells, we then focused on characterizing CA in these cells. To provide additional evidence supporting CA inhibition of mTORC1, we examined ULK1, another mTORC1 substrate whose phosphorylation at Ser757 by mTORC1 also serves as a readout for mTORC1 activity (Kim et al., 2011; Klionsky et al., 2021). Similar to the case with S6K, CA treatment inhibited ULK1 phosphorylation in HEK293T cells (Figure 6c,d), providing additional evidence supporting that CA can inhibit mTORC1 activity in HEK293T cells. These results are consistent with the observations that CA promotion of longevity was severely defective in *atg‐1/ULK1* mutant worms (Figure 3f), indicative of the involvement of ATG1/ULK1 in CA action. We also tested AITC, a potent agonist of TRPA1 channel (Bandell et al., 2004; Jordt et al., 2004), and found that AITC failed to inhibit mTORC1 activity in HEK293T cells (Figure S6a–d), congruent with our *C. elegans* data showing that the action of CA is independent of TRPA‐1 (Figure S1). These results together demonstrate that CA can inhibit mTORC1 activity in human cells.

Inhibition of mTORC1 is known to trigger autophagy (Deleyto‐Seldas & Efeyan, 2021; Kim & Guan, 2019; Saxton & Sabatini, 2017; Wullschleger et al., 2006). We then assessed the effect of CA on autophagy by monitoring the LC3‐II and SQSTM1 levels, an assay commonly employed to determine autophagic activity (Klionsky et al., 2021). CA treatment led to an increase in the LC3‐II level as well as the SQSTM1 level in the presence of lysosomal proteinase inhibitors (E‐64d and leupeptin) in HEK293T cells (Figure 6e–h; Figure S5; Figure S6e–h), suggesting that CA enhanced autophagic flux. This is consistent with the observation that CA inhibited the phosphorylation of ULK1 at Ser757 (Figure 6c,d), which is expected to trigger the initiation of autophagy and its activity (Hurley & Young, 2017). This is also consistent with our data in *C. elegans* showing that CA promoted autophagy (Figure 3i,j) and that CA promotion of lifespan and healthspan required mTORC1 and autophagy signaling (Figure 3; Figure 4). Thus, CA appears to regulate mTORC1‐autophagy signaling in human cells, suggesting that the mode of action of CA might be conserved in worms and mammals.

## 3 DISCUSSION

As one of the most popular spices, cinnamon has been utilized for thousands of years, dating back to ancient Egyptian times (Otness, 1998; Toussaint‐Samat, 2009). In addition, the discovery of the medicinal properties of cinnamon has further expanded its use (Blaszczyk et al., 2021). However, the lack of an understanding of the mechanisms governing the impact of cinnamon on physiology and pathophysiology has greatly limited its application. In the present study, we found that CA, the active ingredient in cinnamon oil, can delay aging by promoting both lifespan and healthspan in *C. elegans* (Figure 6i). To the best of our knowledge, this represents the first report of a role of cinnamon in aging. In addition, CA can ameliorate β‐Amyloid toxicity in a *C. elegans* model of AD. More importantly, we show that CA acts through mTORC1‐autophagy signaling, identifying a target of CA. Strikingly, CA can also regulate mTORC1‐autophagy signaling in human cells. A shared mechanism of action of CA in both worms and human cells raises the possibility that CA may exert a similar effect on aging and aging‐related diseases in higher organisms.

Cinnamon has long been recognized for its therapeutic potential for various human diseases, ranging from diabetes and cancer to neurodegenerative diseases (Banerjee & Banerjee, 2023; Medagama, 2015; Momtaz et al., 2018; Shang et al., 2021; Silva et al., 2022). Such a wide spectrum of health benefits of cinnamon invites the question of whether these effects are specific, and if so, what specific physiological processes cinnamon targets. Our observation that CA regulates mTORC1‐autophagy signaling provides a mechanistic understanding of the action of cinnamon. Nevertheless, our work does not necessarily indicate that mTORC1‐autophagy signaling is the only target of cinnamon. For example, cinnamon's anti‐bacterial property likely involves a different mechanism, as mTORC1‐autophagy signaling is absent in prokaryotes (Wullschleger et al., 2006). Additionally, as higher concentrations of cinnamon appeared to be toxic to worms, it may recruit additional targets or act through completely different mechanisms (Lu et al., 2020; Ropiak et al., 2016). We thus do not exclude the possibility that additional cinnamon targets might exist, probably in both prokaryotes and eukaryotes.

As mTORC1‐autophagy signaling plays a central role in metabolism and cellular homeostasis (Kim & Guan, 2019; Saxton & Sabatini, 2017; Wullschleger et al., 2006), it has been widely harnessed to develop therapeutics to treat a growing list of human diseases (Aman et al., 2021; Deleyto‐Seldas & Efeyan, 2021; Saxton & Sabatini, 2017). For example, rapamycin and its derivatives, which are potent mTORC1 inhibitors, have shown great potential in treating cancer, diabetes, and neurodegenerative diseases, and can also promote longevity in various animal models (Kim & Guan, 2019; Saxton & Sabatini, 2017). However, rapamycin is associated with severe side effects. For example, chronic inhibition of mTOR by rapamycin induces insulin resistance and triggers immunosuppression due to its block of mTORC2 in addition to mTORC1 (Saxton & Sabatini, 2017). This has greatly limited its applications in the clinics. In *C. elegans*, chronic rapamycin treatment can also block both mTORC1 and mTORC2 (Robida‐Stubbs et al., 2012), and the longevity‐promoting effect of rapamycin depends on SKN‐1/Nrf2 (Blackwell et al., 2015; Robida‐Stubbs et al., 2012). By contrast, CA promotion of longevity does not require SKN‐1/Nrf2, but instead depends on PHA‐4/FOXA and HLH‐30/TFEB. This might result from the fact that unlike rapamycin, CA promotes longevity in an mTORC1‐dependent but mTORC2‐independent manner. Thus, while CA and rapamycin both inhibit mTORC1, their modes of action are not identical. Though the exact mechanisms underlying CA inhibition of mTORC1 are currently unclear, our finding that CA acts through mTORC1 but not mTORC2 presents a potential advantage in the development of cinnamon‐based therapeutics.

One interesting observation is that dietary CA and DR appear to converge on the mTORC1‐autophagy signaling to regulate aging (Figure 6i). Consistent with this model, transcriptomic analysis reveals a substantial overlap of pathways (GO terms) and DEGs induced by CA and DR. While this by no means demonstrates that the transcriptomes of worms fed CA and under DR are identical, they bear clear similarities, suggesting that dietary CA may induce a DR‐like state in worms. As CA does so without affecting food intake, this points to the possibility that CA may act as a DR mimetic agent. On the other hand, CA treatment increased lipid storage in *C. elegans* (Figure S1j), indicative of a complex role of CA in longevity and metabolism. Notably, cinnamon has also been credited for its potential to combat obesity (Blaszczyk et al., 2021; Keramati et al., 2022; Zuo et al., 2017). Specifically, cinnamon supplementation can reduce body weight and body mass index (BMI) in human studies (Keramati et al., 2022). However, the mechanistic basis of such anti‐obesity properties of cinnamon is unknown. Our observation that CA may act as a DR memetic provides mechanistic insight into this feature of cinnamon. Our work will inspire and guide the further development of cinnamon as both a therapeutic and pro‐health agent.

## 4 MATERIALS AND METHODS

### 4.1 *C. elegans* strains and maintenance

*C. elegans* strains were maintained at 20°C on nematode growth medium (NGM) plates seeded with *E. coli* OP50 bacteria unless otherwise specified. For paralysis assays, worms were kept at 15°C as recommended until use. The wild‐type strain: N2. Strains used in this study were: TQ3030 N2, TQ1516 *trpa‐1(ok999)*, TQ7760 *smg‐1(cc546)*; *dvIs27 Pmyo‐3::A‐Beta(1–42)::let‐851 3UTR + rol‐6(su1006)*, TQ6018 *adIs2122 [lgg‐1p::GFP::lgg‐1 + rol‐6(su1006)]*, TQ6810 *eat‐2(ad465)*, TQ1602 *daf‐2(1368)*.

### 4.2 Chemical reagents

trans‐CA (#C80687), 5‐Fluoro‐2′‐deoxyuridine (FUDR, #F0503), 3‐Indoleacetic acid (IAA, #I2886), E‐64d (#E8640), Leupeptin (#L2884), Latex beads (#L3280), tBHP (#458139), Pierce Phosphatase Inhibitor (#A32957) were acquired from Sigma Aldrich. Protease inhibitor cocktails (#11836153001) were acquired from Roche. LC3 Polyclonal antibody (#4600‐1‐AP), P62/SQSTM1 Polyclonal antibody (#18420‐1‐AP) were purchased from Proteintech. S6K antibody (#2708), phospho‐S6K antibody (#97596), ULK1 antibody (#8054), and phosphor‐ULK1 antibody (#6888) were purchased from Cell Signaling. 4x Laemmli Sample Buffer (#1610747) were obtained from Bio‐rad.

### 4.3 Lifespan assays

All lifespan assays were performed at 20°C. Age‐synchronized worms were acquired from gravid worms laying eggs for 4 h on standard NGM plates. About 100 synchronized L4 worms were transferred onto NGM plates seeded with OP50 bacteria containing indicated concentrations of CA (25 μM for most cases). Worms were transferred every other day to a fresh plate containing indicated concentrations of CA until day 12, at which point CA concentration was increased to a higher level (either 100, 200, or 300 μM). For RNAi experiments, L1/2 larval worms were transferred onto NGM plates seeded with OP50(xu363) RNAi bacteria containing CA, except for *bec‐1* and *lgg‐1*, in which case HT115 RNAi bacteria were used (Xiao et al., 2015). In all experiments, the first day of adulthood was scored as day 1. Animals that crawled off the plate, exploded or bagged were censored. Animals that did not respond to mechanical prodding were scored as dead. Survival of the animals was examined every 2 to 4 days.

For bacterial dietary restriction (bDR), the lifespan assays were performed as described in previous literature with modification (Panowski et al., 2007). bDR media were prepared as below. To harvest bacterial food, fresh OP50 colonies were inoculated in LB medium overnight (14–16 h), and bacterial pellets were collected by centrifugation (4000 rpm, 4°C, 20 min) and washed twice with S Basal, then resuspended in fresh S medium containing 100 μg/mL carbenicillin to a final concentration of 10^10^ cfu/mL. Carbenicillin treatment stopped OP50 growth, but this does not necessarily indicate that these OP50 bacteria were metabolically inactive. Ad libitum (AL) food was prepared with a bacteria concentration of 10^10^ cfu/mL, and DR food with a bacteria concentration of 0.2 × 10^10^ or 1 × 10^9^ cfu/mL (5× and 10× dilution, respectively). 5‐Fluoro‐2′‐deoxyuridine (FUDR) was added to food solution at a final concentration of 25 μg/mL to prevent egg‐laying and bagging. Synchronized L4 worms were transferred onto NGM plates seeded with OP50 containing 25 μM CA for 24 h, then transferred onto NGM plates seeded with OP50 containing 25 μM CA and 2.5 μg/mL FUDR for another 24 h. On day 2, worms were transferred to a small petri dish with 3 mL S medium solution containing 100 μg/mL carbenicillin and 25 μg/mL FUDR with or without 10 or 50 μM CA for 1 h to remove any residual OP50 bacteria clumps. Finally, 15 worms per well were moved to a 12‐well cell culture plate with 1 mL/well food solution prepared as mentioned above with or without 10 or 50 μM CA and placed on a 20°C shaker with a speed of 90 rpm throughout the experiment. Food solutions were refreshed twice per week, at which time point worms were scored for movement with eyelash prodding, and any non‐moving worms were scored as dead and abandoned. FUDR was added during the first 2 weeks of the experiment. About 60 worms were used for each treatment.

All mock treatments referred to the use of an equal volume of solvent (ethanol) without CA. All statistical analyses were performed using GraphPad Prism 8 (GraphPad Software, Inc.) and IBM SPSS Statistics 21 (IBM, Inc.). *P* values were calculated using the log‐rank (Kaplan–Meier) method.

### 4.4 Chemotaxis assays

Chemotaxis assays were modified from previously described methods (Bargmann et al., 1993). Test plates for chemotaxis were made using 10 cm petri dishes. Well‐fed day 1 adult animals were collected and washed with S basal buffer (5.85 g NaCl, 1 g K_2_HPO_4_, 6 g KH_2_PO_4_, H_2_O to 1 L) for three times. Fifty to one hundred adult worms were tested for each plate. Chemoattraction to the odorant was performed by placing worms near the center of the plate at a point equidistant to the point source attractant (10 μL diluted CA in DMSO) and the counter attractant (10 μL DMSO). 2 μL 500 mM sodium azide was also spotted at the attractant and counter attractant points just prior to testing to paralyze worms that reached the point sources. The assay ran for 1 h at 20°C in the dark and then moved to 4°C to paralyze worms until scoring. The chemotaxis index was calculated as:

[N_(attractant)_‐N_(counterattractant)_]/[N_(attractant)_ + N_(counterattractant)_]

Chemotaxis assays were repeated at least three times for each experimental condition.

### 4.5 Brood size quantification and oil red O staining

For brood size assay, synchronized L4 animals were picked individually onto NGM plates seeded with fresh OP50 bacteria and allowed to lay eggs for 24 h at 20°C. They were then moved to fresh plates every 24 h for the duration of the reproduction active phase for at least 5 days. The progenies were allowed to grow up at 20°C for 2 days, after which the number of animals was manually counted. Experiments were repeated at least three independent times.

For Oil Red O staining and quantification, all experiments were conducted at 20°C. Animals were treated with different concentrations of CA from L4. On day 3 of adulthood, worms were collected and washed twice with S basal buffer, followed by resuspension in 60% isopropanol for 15 min for dehydration. After all isopropanol was removed, worms were incubated in 500 μL ORO staining solution prepared as follows: 0.5 g ORO was dissolved in 100 mL isopropanol and equilibrated for 2 days by stirring to make ORO stock solution. ORO staining solution was freshly prepared by mixing 60% ORO stocking solution with 40% distilled water and filtered through a 0.2 μm syringe filter overnight, and then saved in 500 μL S buffer containing 0.01% Triton X‐100 from the staining solution for imaging. For imaging and lipid content quantification, animals were mounted, and images were acquired through an Olympus BX53 microscope coupled with a digital camera, followed by background subtraction and conversion into gray scale using ImageJ. Signal intensities were then acquired and compared among worms treated with different conditions. Experiments were repeated at least three independent times. Significance was determined using one‐way ANOVA with Dunnett's test.

### 4.6 Pumping rate and food ingestion measurements

L4 worms were transferred to NGM plates seeded with live or carbenicillin‐treated bacteria food (concentrated to 10^11^ cfu) with or without CA and then moved to corresponding plates with 2.5 μg/mL FUDR at day 1 and day 3 to remove progeny. Pharyngeal pumping rate was then quantified under a stereoscope.

To quantify food ingestion, at the time point of measurements (day 2, day 5, and day 7), worms were transferred to corresponding fresh plates containing carboxylate‐modified polystyrene latex beads (fluorescent red). One hour prior to the experiment, the beads were mixed with OP50 bacteria (1:100 dilution) and seeded on NGM plates. Then worms were immobilized in 20 mM sodium‐azide/M13 solution and mounted on 2% agarose pads, and fluorescence was visualized under an Olympus confocal microscope with a 10× objective. All worms were allowed to crawl on empty NGM plates with no food for 1 min to remove residual bacteria and beads, and imaging was finished within 15 min to prevent defecation. Z stack images of the intestine were captured, and maximum‐intensity Z‐projection images were used for quantification. Fiji (ImageJ) software was used to process the images.

### 4.7 LGG‐1::GFP puncta quantification

*adIs2122* worms were treated with either control or 25 μM, 100 μM CA from hatching. Ten to fifteen L3/L4 larval stage animals were immobilized in 20 mM sodium‐azide/M13 solution and mounted on 2% agarose pads, and fluorescence was visualized under an Olympus confocal microscope with a 40X objective. Z stack images of hypodermal seam cells were captured, and maximum‐intensity Z‐projection images were used for quantification. Fiji (ImageJ) software was used to process the images. GFP::LGG‐1 puncta were quantified by manual counting. Experiments were performed at least three independent times (Kang et al., 2007).

### 4.8 Oxidative stress resistance and thermotolerance assays

For oxidative stress resistance assay, Day 2 adult worms were transferred to NGM plates with 9.125 mM tert‐butyl hydroperoxide with 30 worms per plate. 100 μM CA was used if not specified otherwise. Survival was scored every 2 h until all animals died. Each experiment was repeated at least 3 independent times.

For thermotolerance assay, Day 2 adult worms (20 worms per plate, 5 plates total per condition) were exposed to 34°C in an incubator for 4–8 h. Plates were then removed from the incubator and recovered at room temperature for 12 h. The survival rate was scored for each treatment. Each experiment was repeated at least 3 indpendent times.

### 4.9 Locomotion assay

Locomotion assay was performed using a WormLab system (MBF Bioscience). A 20 μL freshly grown OP50 bacteria was spread as a thin layer on assay plates containing CA 20 min before recording. 4–6 worms were placed on assay plates and habituated 10 min before recording. Videos were recorded for 1 min for each worm. For analysis, those few worms with no motor activity for more than 30 s were censored and not included in the analysis. The whole 1 min video clips were used for analyzing locomotion speed and worm body length using software from WormLab. Locomotion assays were repeated at least three times with 10–18 worms each time for each experimental condition. At least 30 worms were assayed in total.

### 4.10 Paralysis assay

Worms expressing β‐Amyloid protein in muscles (*dvIs27*) were used in this assay. Worms were raised until the L4‐young adult stage at 15°C, after which they were shifted to NGM plates seeded with OP50(xu363) RNAi bacteria with CA and 2.5 μg/mL FUDR at 20°C for the assay. About 30 worms of each treatment were monitored every other day throughout the whole life. Paralysis was scored when animals did not move their body after stimulation with platinum pick prodding on the nose. Paralysis assays were repeated at least three times for each experimental condition (Link, 1995). *P* values were calculated using the log‐rank (Kaplan–Meier) method.

### 4.11 RNA‐seq and data analysis

For sample preparation, animals were divided into four groups: AL‐fed condition (10^10^ cfu) with or without 50 μM CA, and DR‐fed condition (10^9^ cfu) with or without 50 μM CA. About 240 worms were treated as described in the lifespan assay and were incubated in liquid medium for 2 days and harvested at Day 4 adult stage. Biological replicates were prepared on separate days. Worms were collected and washed twice with M9 buffer to remove eggs or residual bacteria, and total RNA of each treatment was extracted with TRIzol LS Reagent (Invitrogen) by chloroform/phenol extraction followed by isopropanol precipitation and two washes with 75% ethanol before resuspension in RNase‐free water. RNA samples were further treated with DNase I and cleaned using RNeasy MinElute Cleanup Kit (Qiagen). Quality controls, preparation of cDNA, library construction, and sequencing were performed by the Advanced Genomics Core at the University of Michigan. The library pool was subjected to 151 bp paired‐end sequencing according to the manufacturer's protocol (Illumina NovaSeq).

For RNA‐seq data analysis, the raw paired‐end reads were trimmed using Cutadapt v2.3 and then mapped to the reference genome WBcel235 (ENSEMBL), using STAR v2.7.8a and assigned count estimates to genes with RSEM v1.3.3. The remaining analysis was performed with Bioconductor implemented in R statistical environment (v 4.1.3). Differentially expressed genes were identified using the DESeq2 tool with a threshold of absolute fold‐change greater than 1.5‐fold and a false discovery rate (FDR) smaller than 0.05. Principal Component Analysis (PCA) was used to evaluate the quality of samples and comparisons. Rlog transformation in DESeq2 package was used to generate log2 scale data to represent expression levels for hierarchical clustering. Gene Ontology analysis was performed using Enrichment Analysis tool in Wormbase with a q‐value threshold of 0.1.

### 4.12 Cell culture

HEK293T cells and HeLa cells were cultured in DMEM medium containing 10% heat‐inactivated fetal bovine serum in a 37°C incubator containing 5% CO2. Both the HEK293T cell line and HeLa cell line were obtained from the ATCC. We note that different sources of HEK293T and HeLa cells exhibit different sensitivities to CA. Cells were loaded into a 12‐well plate (1 mL medium per well). Before the treatment, the culture medium was replaced with a fresh one, either with or without the chemical of interest (rapamycin/CA/AITC). Cells were then incubated overnight (8–12 h) before harvesting. For the detection of autophagy‐related proteins (LC3 and SQSTM1/p62), 2 h before harvesting, autophagy inhibitors (E‐64d and leupeptin) were added to the medium at a final concentration of 10 μM and 100 μM, respectively.

Cells were lysed in ice‐cold RIPA buffer (150 mM NaCl, 50 mM pH 8.0 Tris–HCl, 1% NP‐40, 0.5% sodium deoxycholate, 0.1% SDS) supplemented with a protease inhibitor cocktail and a phosphatase inhibitor tablet without PBS wash, as the activity of mTORC1 is sensitive to cold PBS wash (Hong et al., 2012). Cell samples were briefly sonicated with ultrasound followed by centrifugation. The supernatant was mixed with sample buffer (Bio‐rad) and boiled for 5 min. Proteins were separated by SDS‐PAGE and transferred to PVDF membranes. The blots were then processed for Western.

### 4.13 Statistical analysis

Samples were randomized and treated under the same conditions. Data collection and analysis were not performed blindly. Statistics were performed using IBM SPSS Statistics for lifespan and healthspan assays and using GraphPad Prism for other assays. The number of biological independent experiments was indicated in S1 Table for lifespan and healthspan assays. For other assays, experiments were repeated independently at least three times with similar results, and the statistical parameters, error bars, and n‐number were all described in the figure legends or methods described above. Specifically, for those involving multi‐groups comparisons, we applied ANOVA. For those involving lifespan and healthspan data, we applied the log‐rank (Kaplan–Meier) test.

## AUTHOR CONTRIBUTIONS

Y.G. and Q.Z. performed the experiments and analyzed the data. B.Z., T.P., E.A.R. and K.I. assisted Y.G. with experiments and data analysis. A.H. and Y.H. assisted Y.G. with data analysis. Y.G., J.F. and X.Z.S.Z. wrote the paper with help from all other authors.

## CONFLICT OF INTEREST STATEMENT

The authors declare no competing interest.

## Supporting information


Table S1.

Table S2.

Data S1.

## Data Availability

All data supporting the findings of this study are available within the paper and its Supplementary Information. The sequencing data used in this study are available on request from the authors.
