# Supplementary material for: Dietary cinnamon promotes longevity and extends healthspan via mTORC1 and autophagy signaling
Source: Aging Cell. 2025 Jan 6;24(4):e14448. doi: 10.1111/acel.14448 (PMC11984692; doi:10.1111/acel.14448)
Supplement: Supplementary file 3 — Data S1. [file ACEL-24-e14448-s003.pdf]

Supplementary Figure 1

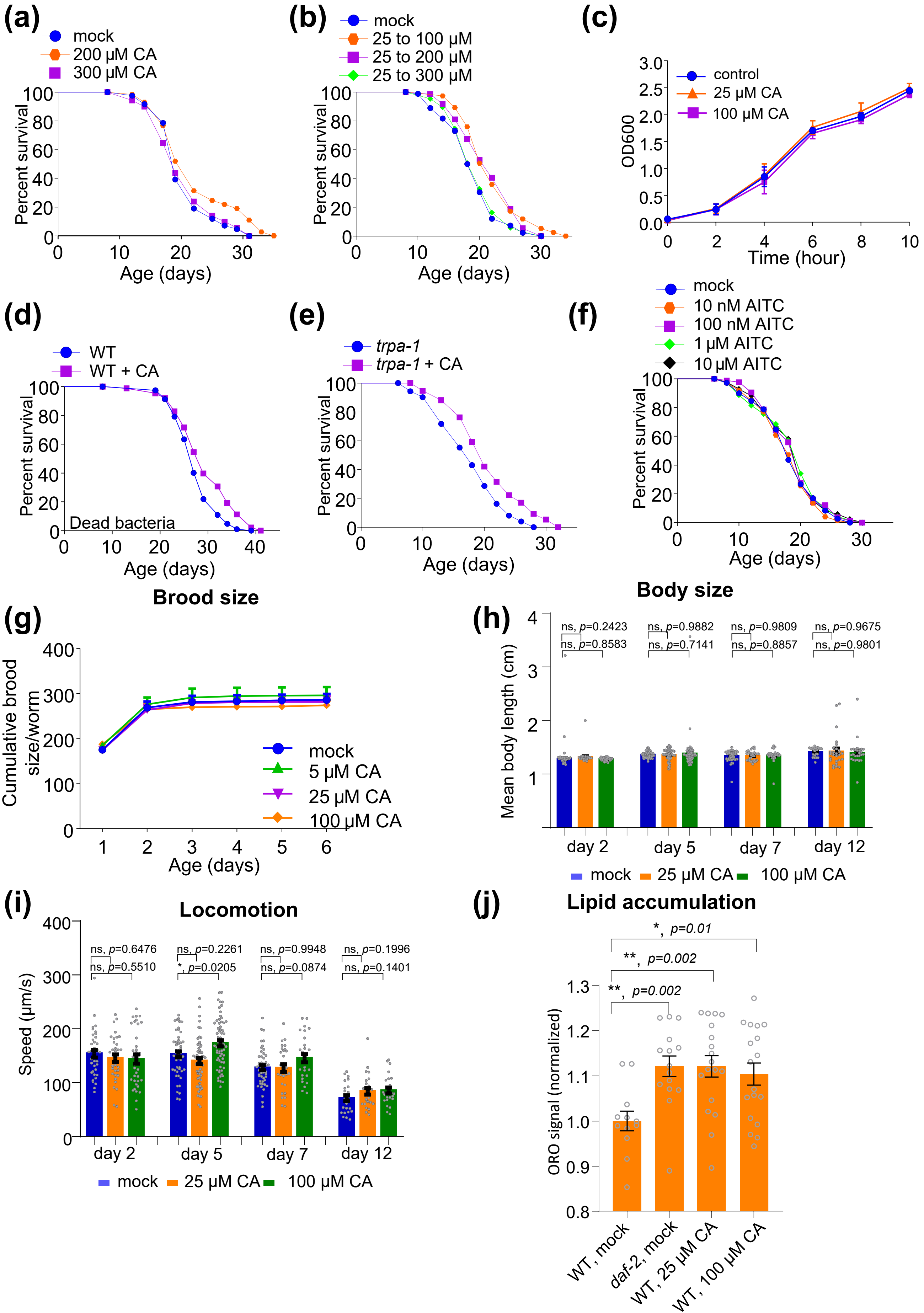

Figure S1. CA does not notably alter the growth rate of OP50 bacteria, and CA-induced lifespan extension is independent of TRPA1. (a) Higher concentrations (e.g. 300 μM) of CA do not extend lifespan. (b) Optimization of the CA lifespan assay. (c) CA does not change the growth rate of OP50 bacteria. The curve depicted bacteria optical density. OD600 values of bacteria cultured with CA were measured at the indicated time points. Data is presented as mean ± SEM. (d) Lifespan survival curve showing that CA can extend the lifespan of worms fed dead bacterial food ($p = 0.002$). OP50 was killed by carbenicillin. (e-f) CA-induced longevity is independent of TRPA-1. (e) Lifespan curve showing that CA still extends lifespan in *trpa-1(ok999)* mutant worms ($p < 0.001$), WT control was shown in Table S1. (f) AITC, an agonist of TRPA1, does not extend lifespan in C. elegans ($p = 0.269$ for 10 nM, 0.651 for 100 nM, 0.125 for 1 μM, 0.589 for 10 μM, respectively, all against mock control). Worms were treated with 25 μM CA from the L4 stage and transferred every other day until day 12, at which point CA concentration was increased to 100 μM and no more progeny was produced. See Methods for details. Kaplan-Meier survival analysis with log-rank test was used for statistical analysis. See Table S1 for lifespan statistics. (g) Dietary cinnamon does not affect brood size. The curve depicted the average brood size (total numbers over days) of worms that fed various concentrations of cinnamon (5 μM, 25 μM, 100 μM). The numbers of offspring on each day from day 1 to day 6 were counted. Data is presented as mean ± SEM. (h) Dietary cinnamon does not affect the body size of worms. Data is presented as mean ± SEM. Experiments were repeated at least three times. p values were calculated using one-way ANOVA with Dunnett's test.(i) Dietary cinnamon does not grossly affect locomotion. Bar graph showing the average speed of worms in one minute's recording. Data is presented as mean ± SEM. Experiments were repeated at least three times. p values were calculated using one-way ANOVA with Dunnett's test. (j) Dietary cinnamon enhances lipid accumulation in worms. *daf-2(e1368)* worms, which showed increased lipid storage, were used as a positive control. Bar graph showing Oil Red O staining of day 3 worms treated with CA from the L4 stage. Data is presented as mean ± SEM. Experiments were repeated at least three times. p values were calculated using one-way ANOVA with Dunnett's test.

Supplementary Figure 2

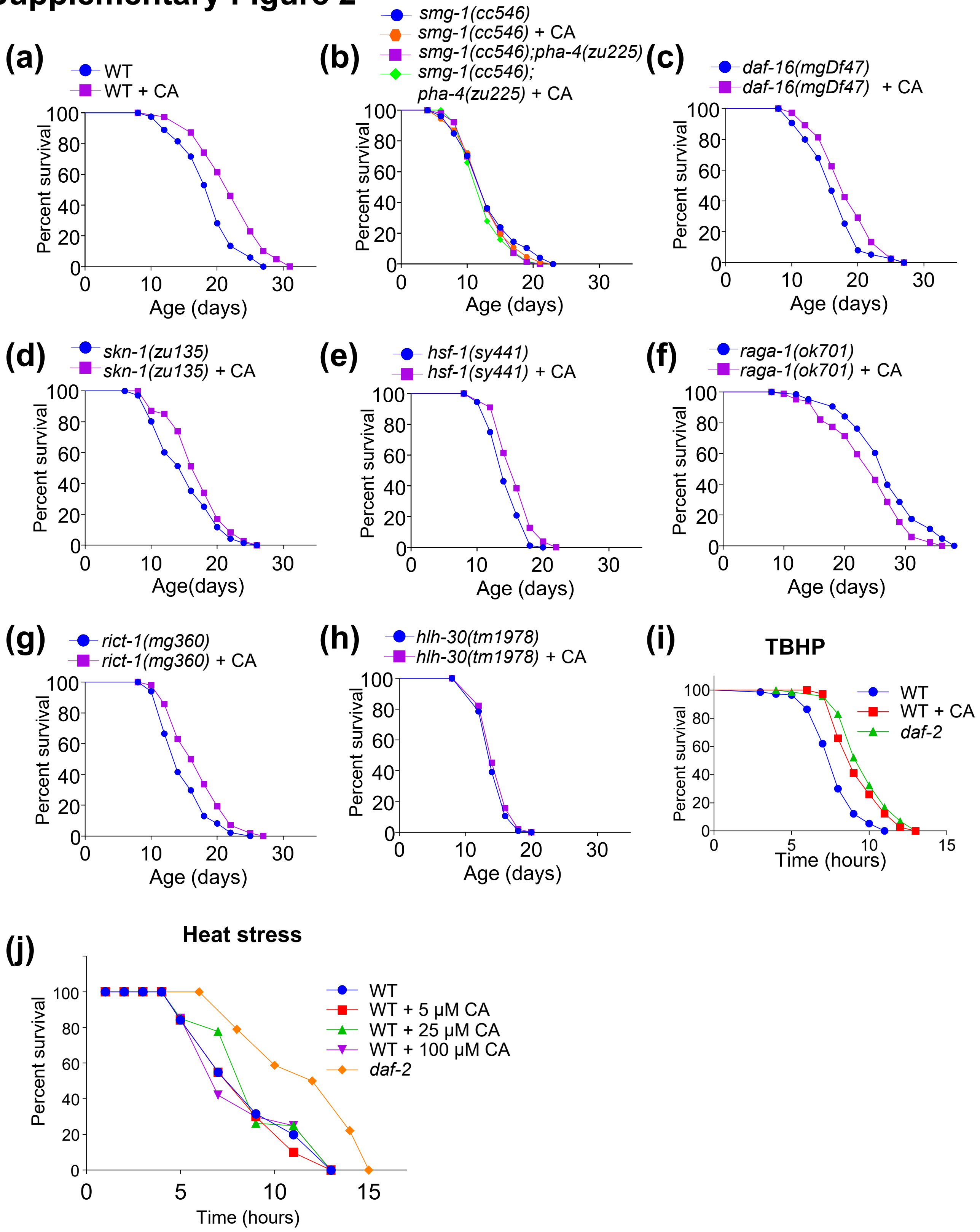

Figure S2. Dietary CA extends lifespan and healthspan via mTORC1 and autophagy signaling. (a) CA extends lifespan in wild type control worms, $p < 0.001$. (b) CA does not extend the lifespan of *smg-1(cc546); pha-4(zu225)* mutant worms ($p = 0.684$). *smg-1* suppressed *pha-4* lethality. This data shall be interpreted with caution as *smg-1* mutant worms were insensitive to CA ($p = 0.465$), likely due to the fact that *smg-1* is part of the mTOR complexes (Wormbase), which are required for CA longevity. The lifespan was performed at 25 ℃ to suppress *pha-4* lethality. (c-e) CA extends lifespan in mutant worms of *daf-16(mgDf47)* ($p = 0.006$) (c), *skn-1(zu135)* ($p = 0.05$) (d), or *hsf-1(sy441)* ($p = 0.001$). (f, g) Inhibition of mTORC1 signaling abolishes CA-induced longevity. Mutations in *raga1(ok701)* ($p = 0.005$) (f), but not *rict-1(mg360)* ($p < 0.001$) (g), abolished the lifespan extension effect of CA. (h) Inhibition of autophagy signaling abolishes CA-induced longevity. Mutations in *hlh-30 (tm1978)* ($p = 0.258$) abolished the lifespan-extension effect of CA. Worms were treated with 25 μM CA from the L4 stage and transferred every other day until day 12, at which point CA concentration was increased to 100 μM and no more progeny was produced. See Methods for details. Kaplan-Meier survival analysis with log-rank test was used for statistical analysis. (i) Dietary CA enhances oxidative stress resistance in WT worms. *daf-2(e1368)* mutant worms, which showed improved oxidative stress resistance, were used as a positive control ($p < 0.0001$). Time course survival curve of worms that were fed 100 μM CA for two days (L4 to day 2) and then exposed to 9.125 mM TBHP. Survival was scored every two hours. (j) Time course survival curve of worms that were fed various concentrations of CA ($p < 0.0001$ for 5 μM, $p = 0.014$ for 25 μM, $p = 0.002$ for 100 μM, all against WT control) for two days and then exposed to 34 ℃ heat for 4 to 8 hours. *daf2(e1368)* mutant worms, which showed improved heat stress resistance, were used as a positive control ($p < 0.0001$). 100 worms were assayed for each experiment. The survival rate was scored every one to two hours 12 hours after recovery at room temperature. See Table S1 for healthspan statistics.

Supplementary Figure 3

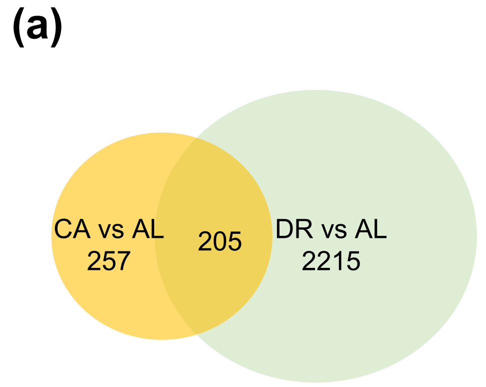

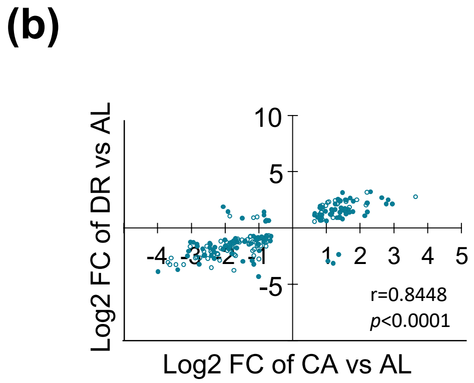

Figure S3. Additional data analysis related to RNA-seq. (a) Pie graph showing the number of genes that are differentially expressed between worms treated with CA (CA vs AL) or worms undergoing dietary restriction (DR vs AL) (log2-fold change > 1.5 and false discovery rate (FDR) < 0.1). (b) Two-axis scatter plot of log2-fold changes showing the expression fold change of the 205 DEGs induced by CA (CA vs AL) and DR (DR vs AL). Each dot represents the expression log2-fold change of a DEG induced by CA (CA vs AL; xaxis value) and by DR (DR vs AL; y-axis value). Pearson correlation coefficient (r) and p value were shown.

Supplementary Figure 4

(a)

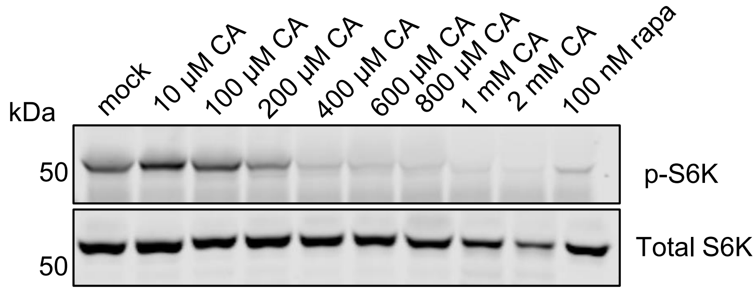

Figure S4. CA inhibits mTORC1 in Hela cells. (a) CA inhibits the phosphorylation of S6K (ribosomal S6 kinase) in HeLa cells in a dose-dependent manner. The working concentration of CA started from 200 μM in Hela cells, which was much higher than that in HEK293T cells, indicating a higher efficacy in HEK293T cells. Rapamycin, an mTORC1 inhibitor, was used as a positive control.

Supplementary Figure 5

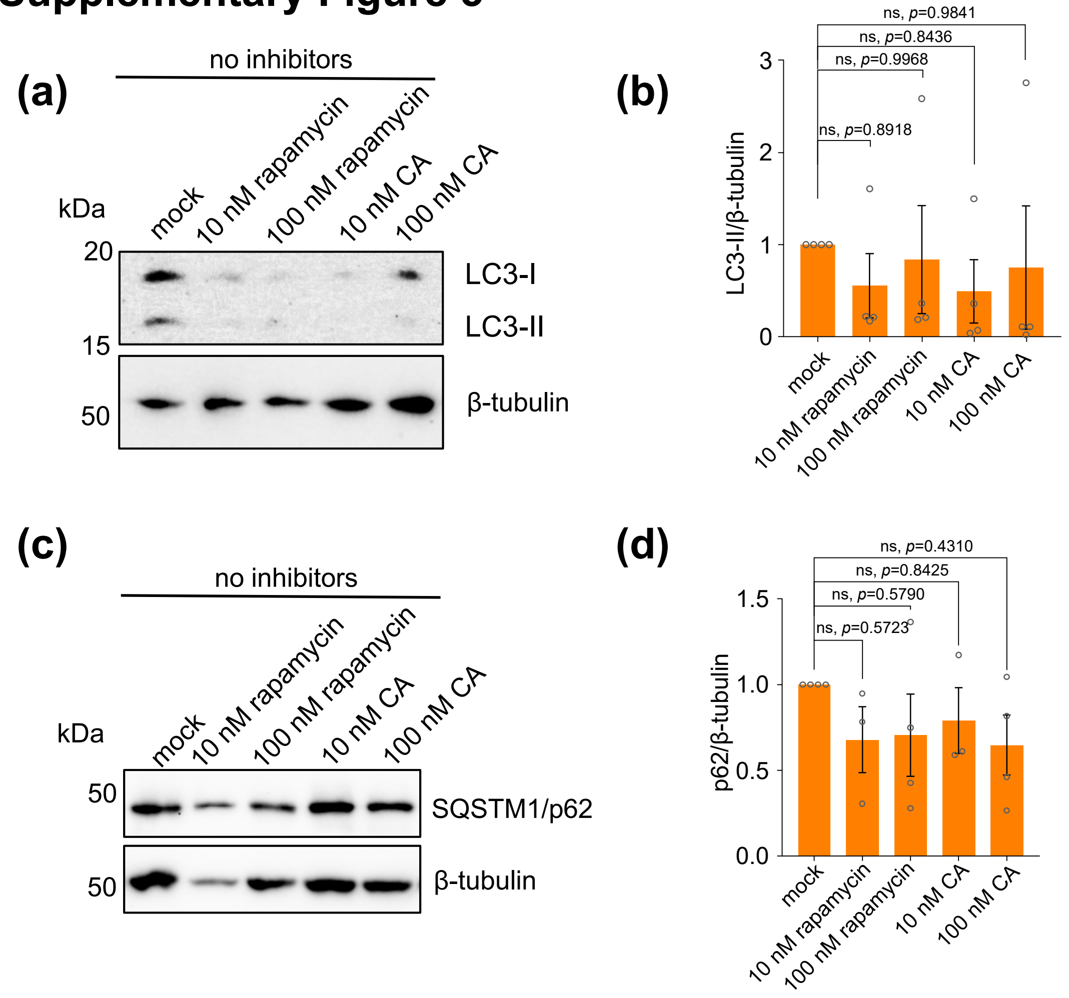

Figure S5. Additional data related to the effect of CA on autophagy in HEK293T cells. (a) Western blotting showing that CA and rapamycin do not increase the amount of LC3-II in the absence of E-64 and leupeptin. (b) Bar graph. Data is presented as mean ± SEM. Experiments were repeated at least three times. p values were calculated using one-way ANOVA with Dunnett's test. (c) Western blotting showing that CA and rapamycin do not increase the amount of SQSTM1/p62 in the absence of E-64d and leupeptin. (d) Bar graph. Data is presented as mean ± SEM. Experiments were repeated at least three times. p values were calculated using one-way ANOVA with Dunnett's test.

Supplementary Figure 6

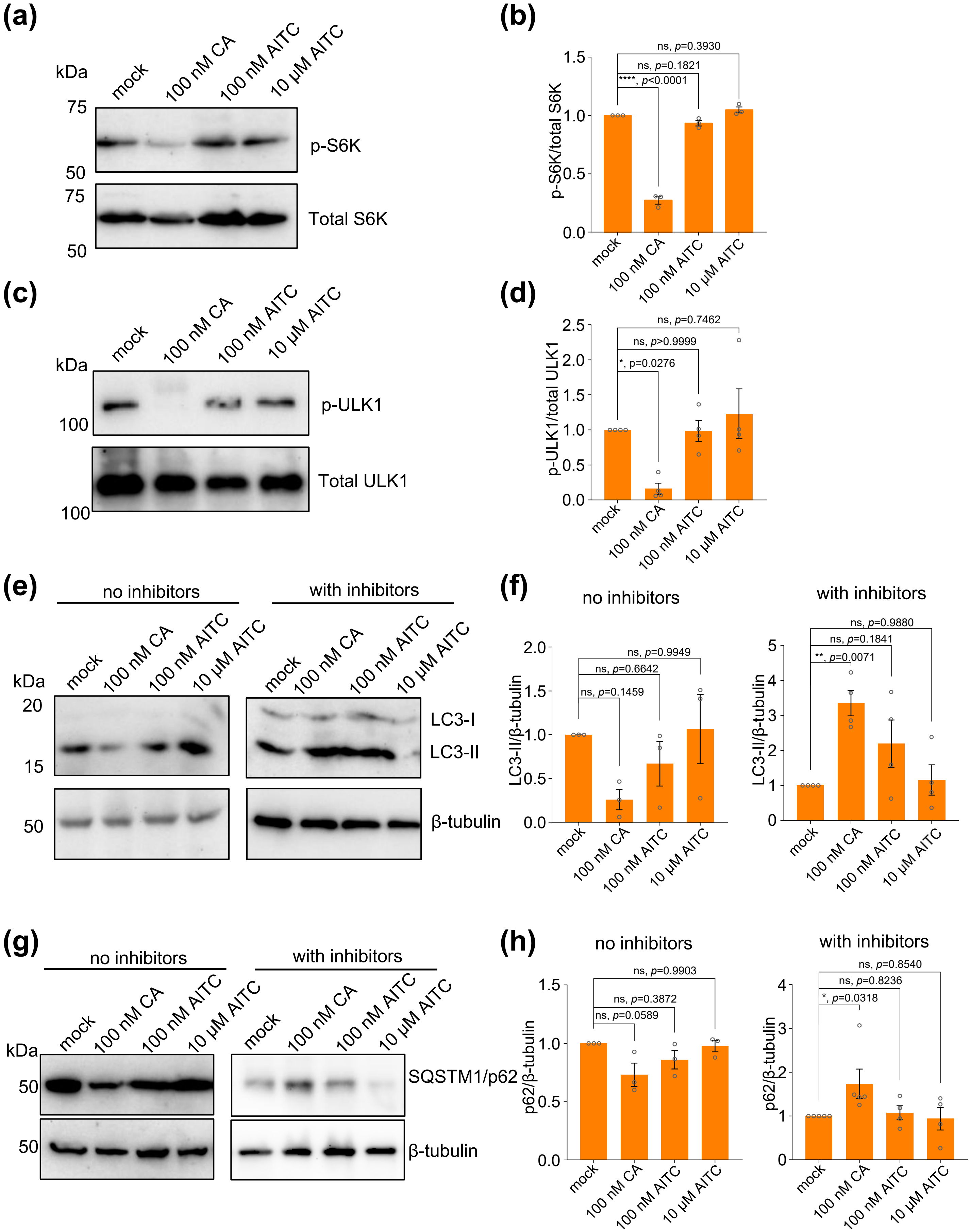

Figure S6. AITC has no influence on the activity of mTORC1 or autophagy in HEK293T cells. Related to Figure 6. (a) Western blotting showing AITC does not inhibit the phosphorylation of Thr389 site in S6K. (b) Quantification bar graph. Data is presented as mean ± SEM. Experiments were repeated at least three times. p values were calculated using one-way ANOVA with Dunnett's test. (c) Western blotting showing AITC does not inhibit the phosphorylation of Ser757 site in ULK1. (d) Quantification bar graph. Data is presented as mean ± SEM. Experiments were repeated at least three times. p values were calculated using one-way ANOVA with Dunnett's test. (e) Western blotting showing AITC does not increase the amount of LC3-II in the presence or absence of E-64 (10 nM) and leupeptin (100 nM). (f) Quantification bar graph. Data is presented as mean ± SEM. Experiments were repeated at least three times. p values were calculated using one-way ANOVA with Dunnett's test. (g) Western blotting showing AITC does not increase the amount of SQSTM1/p62 in the presence or absence of E-64 (10 nM) and leupeptin (100 nM). (h) Bar graph. Data is presented as mean ± SEM. Experiments were repeated at least three times. p values were calculated using one-way ANOVA with Dunnett's test.
